# Low priority of obesity education leads to lack of medical students’ preparedness to effectively treat patients with obesity: results from the U.S. medical school obesity education curriculum benchmark study

**DOI:** 10.1186/s12909-020-1925-z

**Published:** 2020-01-28

**Authors:** W. Scott Butsch, Robert F. Kushner, Susan Alford, B. Gabriel Smolarz

**Affiliations:** 1 Bariatric and Metabolic Institute at Cleveland Clinic, Cleveland, OH USA; 2 Northwestern University, Chicago, IL USA; 3 Novo Nordisk Inc, 800 Scudders Mill Rd, Plainsboro Township, NJ 08536 USA

**Keywords:** Obesity, Medical school curricula, Obesity education, Medical student, Medical school education

## Abstract

**Background:**

Physicians are currently unprepared to treat patients with obesity, which is of great concern given the obesity epidemic in the United States. This study sought to evaluate the current status of obesity education among U.S. medical schools, benchmarking the degree to which medical school curricula address competencies proposed by the Obesity Medicine Education Collaborative (OMEC).

**Methods:**

Invitations to complete an online survey were sent via postal mail to 141 U.S. medical schools compiled from Association of American Medical Colleges. Medical school deans and curriculum staff knowledgeable about their medical school curriculum completed online surveys in the summer of 2018. Descriptive analyses were performed.

**Results:**

Forty of 141 medical schools responded (28.4%) and completed the survey. Only 10.0% of respondents believe their students were “very prepared” to manage patients with obesity and one-third reported that their medical school had no obesity education program in place and no plans to develop one. Half of the medical schools surveyed reported that expanding obesity education was a low priority or not a priority. An average of 10 h was reported as dedicated to obesity education, but less than 40% of schools reported that any obesity-related topic was well covered (i.e., to a “great extent”). Medical students received an adequate education (defined as covered to at least “some extent”) on the topics of biology, physiology, epidemiology of obesity, obesity-related comorbidities, and evidence-based behavior change models to assess patient readiness for counseling (range: 79.5 to 94.9%). However, in approximately 30% of the schools surveyed, there was little or no education in nutrition and behavioral obesity interventions, on appropriate communication with patients with obesity, or pharmacotherapy. Lack of room in the curriculum was reported as the greatest barrier to incorporating obesity education.

**Conclusions:**

Currently, U.S. medical schools are not adequately preparing their students to manage patients with obesity. Despite the obesity epidemic and high cost burden, medical schools are not prioritizing obesity in their curricula.

## Background

Obesity is a major public health threat and leading cause of morbidity and mortality in the United States today [1]. The prevalence of obesity is nearly 40% in U.S. adults, with higher rates among certain minority groups [2]. Despite the health and economic impact of obesity on individuals and society [3, 4], medical students remain inadequately trained in obesity and obesity management. A recent survey of medical students found that understanding genetic and biological factors related to obesity correlated with better counseling skills for patients with obesity [5]. This underscores the need to educate medical students on the disease of obesity to reduce bias and improve patient care.

Little progress has been made to incorporate obesity education into undergraduate medical curricula despite studies describing insufficient medical student training in obesity counseling and medical management of obesity [6–8]. In addition, the 2007 Association of American Medical Colleges (AAMC) call to action report, which concluded that “medical education must assure that future physicians will be better prepared to provide respectful, effective care of overweight and obese patients” and that education on preventing and treating obesity should be included in medical school curricula, has not led to any meaningful change in medical education and training [9]. Several studies have uncovered the paucity of nutrition education, an essential component of comprehensive obesity education, in U.S. medical schools [10, 11]. A recent survey on nutrition education reported students receive an average of 19 h of nutrition education over the course of medical school, most within the pre-clinical years, falling short of the recommended 25-h minimum [11]. In addition, a recent study found a limited number of references to obesity in the United States Medical Licensing Examinations (USMLE). The USMLE focuses on weight-related complications of obesity such as type 2 diabetes rather than the disease of obesity itself [12].

To date, there is no literature describing the state of obesity education in undergraduate medical education. The aim of this study is to report how obesity is currently addressed in the curricula of U.S. allopathic medical schools and provide a benchmark from which we can assess progress toward as well as understand barriers to implementing core competencies in obesity.

## Method

Between July and September 2018, we sent invitations to medical school curriculum deans and administrators at 141 accredited allopathic U.S. medical schools identified from the AAMC membership list to request their voluntary participation in this cross-sectional study consisting of an online survey [13].

Survey participation was limited to allopathic medical schools in the U.S., excluding Puerto Rico. Responses were restricted to one representative per medical school to ensure consistent data and equal representation of each institution. Online searches identified a total of 552 potential respondents within the qualifying medical schools using their title as an indicator of curriculum knowledge, e.g., Dean of Medical Education. Potential respondents received a postal mailing with a letter identifying the study sponsor (Novo Nordisk) and key collaborators (Drs. Scott Butsch and Robert Kushner), study objectives, participation requirements, and a modest prepaid incentive of $50 in the form of a check. At least one follow-up telephone call, fax or email was used to remind non-responders to participate. To participate, respondents had to confirm a current role in undergraduate medical education and knowledge of their four-year curriculum.

The survey instrument was comprised of 33 questions addressing the structure, format, content, and method of education; it included multiple choice, scalar, and numeric text questions. Using a 4-point Likert scale (“great extent”, “some extent”, “very little”, “not at all”), respondents were asked about coverage of topics related to the core obesity competencies established by the Obesity Medicine Education Collaborative (OMEC) [14]. We also asked respondents about their expectations regarding future incorporation of obesity into the curriculum and perceived importance of obesity education. The complete survey is available [see Additional file 1].

### Statistical analysis

We performed descriptive statistical analysis (means, frequencies) using SPSS Statistics for Windows 15.0.1 (SPSS, Chicago, Illinois) and Stata/IC 14/1. Data are presented as number and percentage for categorical variables, and continuous data expressed as the mean ± standard deviation (SD) unless otherwise specified.

## Results

### Characteristics of respondents

Forty medical schools (28.4% of U.S. medical schools) responded and completed the survey. These schools (Table 1) are representative of the universe of medical schools in the U.S. [15] in terms of geographic regions (current distribution: Northeast-28.4%, Midwest-24.1%, South-34.8% and West-12.8%) and public and private (60 and 40%, respectively) funding source. Median time to complete the survey was 9 min. Respondents had nearly 20 years of experience in undergraduate medical education and 75.0% of them were medical school deans of education. More than three-quarters of the respondents were “very familiar” with the four-year curriculum and all except two reported teaching medical students. See Table 1 for sample characteristics.

**Table 1:** Characteristics of 2018 Medical School Curriculum Benchmark Online Survey Respondents (*n* = 40)

Respondents	
Title/Role	*n* (%)^a^
Dean of medical education/curriculum	30 (75.0)
Administrator	4 (10.0)
Course/Curriculum coordinator	2 (5.0)
Curriculum director	2 (5.0)
Other	2 (5.0)
Academic Experience (years)	mean ± SD
Time at current institution	15.3 ± 11.0
Time in current role	6.6 ± 4.4
Time involved in undergraduate medical education	19.7 ± 10.3
Role in Undergraduate Education^b^	*n* (%)
Actively teach medical students	38 (95.0)
Teach a course to medical students	25 (62.5)
Give lectures to medical students	33 (82.5)
Participate as a tutor to medical students	9 (22.5)
Participate as a preceptor in clinic to medical students	16 (40.0)
Other	12 (30.0)
Institutions	
Funding Source	*n* (%)
Public	25 (62.5)
Private	15 (37.5)
Region	*n* (%)
Northeast	9 (22.5)
Midwest	12 (30.0)
South	14 (35.0)
West	5 (12.5)

*SD* Standard deviation

^a^Percentages may not sum to 100% due to rounding

^b^Multi-select response options

### Structure of curriculum

Only 7.5% of medical schools reported offering obesity as a standalone course while 60.0% integrated a few elements of obesity education into a broader clinical nutrition or preventative medicine course. Some medical schools (20.0%) used virtual learning systems, e.g., Nutrition in Medicine™ modules, to provide obesity education. Although only 17.5% of medical schools reported teaching obesity in an outpatient clinical medicine rotation, nearly half (47.5%) reported offering elective shadowing opportunities with non-medical providers such as dietitians and psychologists who treat obesity.

### Content of curriculum

**Fig. 1:**
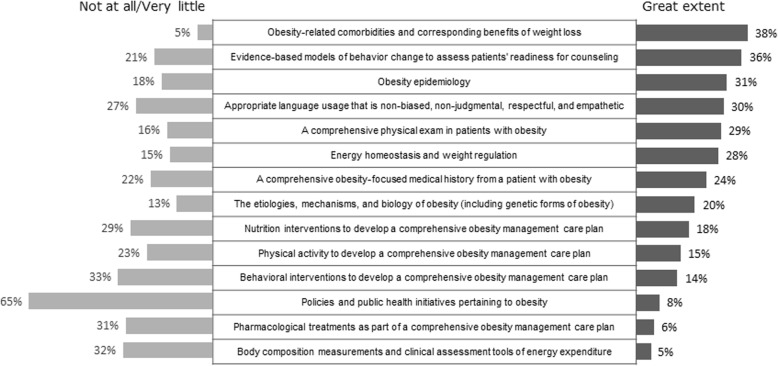
Coverage of Obesity Core Competencies. 2018 Medical School Curriculum Benchmark Online Survey Respondents (*n* = 40). Note: Some competencies have been shortened for presentation. Responses of “Some extent” and “I don’t know” not shown

A mean of ten hours of specific obesity education was taught across the four-year curriculum, however less than 40% of schools reported covering any topic related to core obesity competencies to a “great extent”. Core competencies on basic obesity pathophysiology and the physical examination of a patient with obesity were each covered to a “very little” extent or “not at all” in 15.0% of medical schools. Coverage of non-judgmental communication and use of respectful language with patients who have obesity was covered to a “very little” extent or “not at all” in more than one-quarter of the medical schools. Very few schools thoroughly covered core strategies to develop a comprehensive obesity management care plan such as nutrition interventions, physical activity, behavioral interventions, and pharmacological treatments. Policies and public health initiatives pertaining to obesity were the least covered, with 64.9% of respondents reporting little to no coverage at all (Fig. 1). Schools which reported having an obesity education program currently in place were more likely than those without a program to report most of the core obesity competencies were covered to “some extent” or a “great extent”.

### Preparation of medical students to manage obesity

On a 4-point scale ranging from “not at all prepared” to “very prepared”, only 10.0% of respondents reported that their graduating medical students are “very prepared” to manage patients with obesity; a majority (62.5%) reported that their students are only “somewhat prepared”.

### Priority to incorporate obesity education in curriculum

**Fig. 2:**
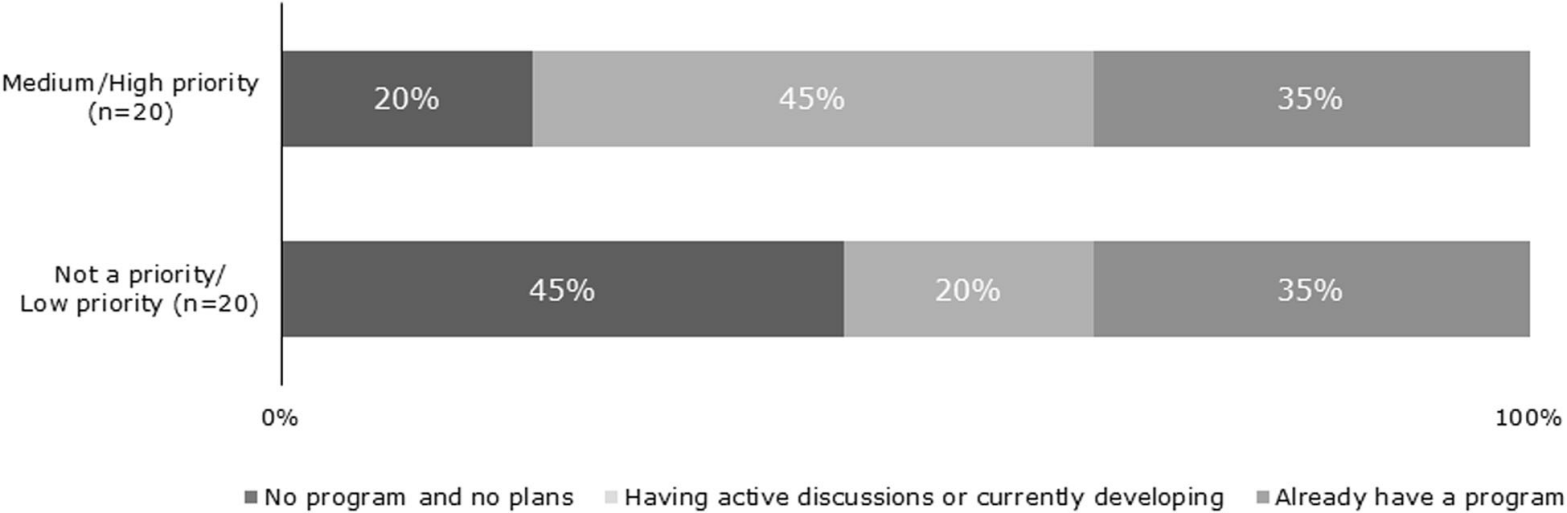
Development of Obesity Curriculum by Stated Priority Level. 2018 Medical School Curriculum Benchmark Online Survey Respondents (*n* = 40)

Expanding obesity education was a low priority or not a priority for 50.0% of those surveyed and nearly half of these schools reported having no obesity education program in place and no plans to develop one. Just over one-third of medical schools reported having an obesity education program in place; of those without a program, only half reported having active discussions on how to incorporate obesity or develop an obesity education program. Of the one-third of medical schools considering incorporating or expanding obesity education, most (76.9%) expected to implement their plans within the next 2 years. Respondents who stated that expanding obesity education is not a priority or is a low priority for their school were less likely to have a program in place or plans to develop one compared to schools in which obesity education is a higher priority (Fig. 2).

**Fig. 3:**
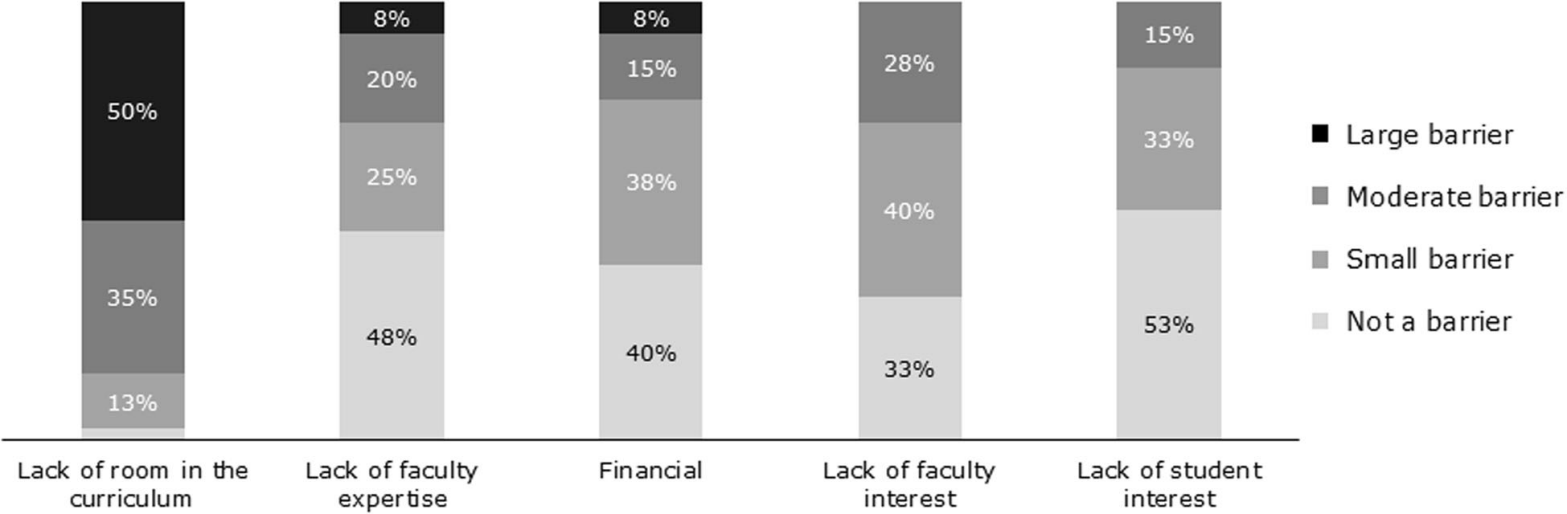
Barriers to Implementing/Expanding Obesity Education in Medical School. 2018 Medical School Curriculum Benchmark Online Survey Respondents (*n* = 40)

*Lack of room in the curriculum* was the most commonly reported barrier to integrating obesity education into the curriculum – half of respondents (50.0%) reported it as a “large barrier” and one-third reported it as a “moderate barrier”. *Lack of faculty expertise* was reported to be a “large” or “moderate” barrier for 27.5% of surveyed medical schools. *Lack of student interest* was least likely to be cited as a barrier. See Fig. 3.

## Discussion

This is the first study to comprehensively assess the state of obesity education in undergraduate medical education in the United States. This Medical School Curriculum Benchmark Study survey revealed inconsistent and inadequate obesity education in U.S. allopathic medical schools resulting in medical students being ill-prepared to manage patients with obesity. Despite the recognition of obesity as a disease by the American Medical Association (AMA) in 2013 and rising prevalence rates of the disease [16, 17], none of the core obesity competencies were well-covered by more than four in ten medical schools surveyed. The survey revealed that in approximately one-quarter to one-third of the medical schools surveyed, there was little to no coverage of rudimentary treatments for obesity, i.e., nutrition, behavioral, and physical activity interventions.

These data underline not only the limited coverage of obesity education, but also the lack of prioritization to develop future curricula in obesity. An overcrowded curriculum was reported as the major barrier to implementing obesity education in this study; however, external barriers, e.g., poor faculty knowledge about obesity, lack of standardized testing on obesity, and overall negative attitudes about the disease of obesity, are possible reasons why obesity education is not prioritized. Nutrition education, one facet of obesity education, is similarly underprioritized in undergraduate medical education. In a recent study of medical student perspectives on why nutrition education is inadequate in medical school, the perception that nutritional care is not the responsibility of doctors was suggested as a barrier [18]. Although our study did not obtain this information, the role of weight bias and the belief that obesity is the result of a voluntary lifestyle choice, and not a biologic disease, may influence decisions and opportunities of inclusion in medical school curricula.

To address the paucity of obesity education in U.S. medical schools, two recent educational initiatives included the development of core competencies in obesity in health care professional schools. First, the Provider Training and Education Workgroup, part of an activity associated with the Roundtable on Obesity Solutions at the National Academies, developed ten high-level provider competencies for the prevention and management of obesity for health care professional schools [19]. Secondly, OMEC, which is spearheaded by the Obesity Medicine Association, The Obesity Society, and the American Society of Metabolic and Bariatric Surgery, developed 32 obesity-related competencies and associated benchmarks across the six core domains of the Accreditation Council for Graduate Medical Education (ACGME). These obesity-related competencies were developed for medical undergraduate and postgraduate training programs to assess learners within a training program [14]. Competencies from both initiatives are the first step to evaluating obesity knowledge of health care professionals and developing a structure for standards of care.

### Limitations

There are several limitations to our study including a response rate of approximately 30%; however, this is not unexpected given the target audience of medical school program leaders who have great demands on their time. To minimize response bias (i.e., inaccurate responses) in our survey, the instrument was designed in collaboration with a survey expert to design optimal questions; however, the survey was not validated, and response bias is possible. Non-responder bias, in which certain types of respondents are less likely to respond (for example, schools without a strong obesity program in place), is also a possibility, and could have skewed the results toward a more favorable outlook of obesity education in U.S. medical schools. We believe that positive skewing is unlikely given the findings of low prioritization of obesity education reported by the respondents. Some of the reported data are subjective, including extent that the obesity competencies are covered, student preparedness, and the prioritization of obesity education.

The design of this research places a greater importance on the number of institutions represented rather than the homogeneity of respondents. It is important to have homogeneity of the respondents, and we believe the deans of education and curriculum leaders were the most appropriate respondents. In our study, nearly all respondents taught medical students and more than 75% were very knowledgeable of their curriculum; however, we were unable to control for the influence of respondents’ varied roles and professional experience on their responses. Additionally, due to the difficulty of true random sampling, this research is limited by the extent to which our sample of 40 schools represents the true population of U.S. allopathic medical schools. Development of the list of contacts was dependent on publicly available information, and therefore, the number of contacts identified at each institution varied. However, our survey sample closely aligned to the composition of the current medical schools in the U.S. with regards to regional distribution and source of funding (public/private). Thus, we believe the sample we obtained represents the population in question (U.S. allopathic medical schools).

## Conclusions

Obesity is a major public health crisis which is clearly not being prioritized within the context of medical school education. Our study highlights the need for U.S. medical school administrators to change their priorities and recognize the urgency to develop curricula that comprehensively address the disease of obesity. Administrators should take advantage of resources provided by organizations such as OMEC and incorporate obesity education into their curricula so that graduating medical students will be more knowledgeable and prepared to address the challenges of caring for and managing the nearly 100 million people with obesity in the U.S. today.

## Supplementary information

**Additional file 1.** Medical School Curriculum Benchmark Survey; Description of data: Survey conducted among medical school directors.

## Data Availability

The datasets used and/or analyzed during the current study are available from the corresponding author on reasonable request.

## Abbreviations

AAMC: Association of American Medical Colleges
ACGME: Accreditation Council for Graduate Medical Education
AMA: American Medical Association
OMEC: Obesity Medicine Education Collaborative
U.S.: United States
USMLE: United States Medical Licensing Examinations

## References

[1] Jia H, Lubetkin EI (2005). The impact of obesity on health-related quality-of-life in the general adult US population. J Public Health (Oxf).

[2] Hales C, Carroll M, Fryar C, Ogden C (2017). Prevalence of obesity among adults and youth: United States, 2015–2016 NCHS data brief, no 288. In.

[3] Finkelstein EA, Trogdon JG, Cohen JW, Dietz W (2009). Annual medical spending attributable to obesity: payer-and service-specific estimates. Health Aff (Millwood).

[4] Wang YC, McPherson K, Marsh T, Gortmaker SL, Brown M (2011). Health and economic burden of the projected obesity trends in the USA and the UK. Lancet.

[5] Fang V, Gillespie C, Crowe R, Popeo D, Jay M (2019). Associations between medical students’ beliefs about obesity and clinical counseling proficiency. BMC Obesity.

[6] Fogelman Y, Vinker S, Lachter J, Biderman A, Itzhak B, Kitai E (2002). Managing obesity: a survey of attitudes and practices among Israeli primary care physicians. Int J Obes Relat Metab Disord.

[7] Metcalf M, Rossie K, Stokes K, Tanner B (2017). The perceptions of medical school students and faculty toward obesity medicine education: survey and needs analysis. JMIR Med Educ.

[8] Vitolins MZ, Crandall S, Miller D, Ip E, Marion G, Spangler JG (2012). Obesity educational interventions in U.S. medical schools: a systematic review and identified gaps. Teach Learn Med.

[9] Association of American Medical Colleges Members Report VIII - contemporary issues in medicine: the prevention and treatment of overweight and obesity. In*.* https://members.aamc.org/eweb/upload/Contemporary%20Issues%20in%20Med%20The%20Prevention%20and%20Treatment%20Report%20VIII.pdf; 2007.

[10] Adams KM, Kohlmeier M, Zeisel SH (2010). Nutrition education in U.S. medical schools: latest update of a national survey. Acad Med.

[11] Adams KM, Butsch WS, Kohlmeier M (2015). The state of nutrition education at US medical schools. J Biomed Educ.

[12] Kushner RF, Butsch WS, Kahan S, Machineni S, Cook S, Aronne LJ (2017). Obesity coverage on medical licensing Examinations in the United States. What is being tested?. Teach Learn Med.

[13] AAMC Medical School Members [https://members.aamc.org/eweb/DynamicPage.aspx?site=AAMC&webcode=AAMCOrgSearchResult&orgtype=Medical%20School].

[14] Kushner RF, Horn DB, Butsch WS, Brown JD, Duncan K, Fugate CS, Gorney C, Grunvald EL, Igel LI, Pasarica M (2019). Development of Obesity Competencies for Medical Education: A Report from the Obesity Medicine Education Collaborative. Obesity (Silver Spring).

[15] Nuttall R (2016). AAMC Organizational Characteristics Database (OCD), July 2015, presented in Medical School Revenues and Budgeting Principles.

[16] AMA Adopts New Policies on Second Day of Voting at Annual Meeting [press release]. Chicago, IL., 2013. [file://kjtfsp01/Shared%20Data/Companywide/Jobs/J19XXX/J19025/References/AMA%20Adopts%20Policy%20to%20Help%20Physicians,%20Students%20Prevent,%20Manage%20Obesity%20_%20American%20Medical%20Association_2017.html].

[17] Revels S, Kumar SAP, Ben-Assuli O (2017). Predicting obesity rate and obesity-related healthcare costs using data analytics. Health Policy Technol.

[18] Mogre V, Stevens FCJ, Aryee PA, Amalba A, Scherpbier AJJA (2018). Why nutrition education is inadequate in the medical curriculum: a qualitative study of students' perspectives on barriers and strategies. BMC Med Educ.

[19] Dietz WH, Gallagher C (2019). A proposed standard of obesity care for all providers and payers. Obesity (Silver Spring).

