# Supplementary material for: Low priority of obesity education leads to lack of medical students’ preparedness to effectively treat patients with obesity: results from the U.S. medical school obesity education curriculum benchmark study
Source: BMC Med Educ. 2020 Jan 28;20:23. doi: 10.1186/s12909-020-1925-z (PMC6988262; doi:10.1186/s12909-020-1925-z)
Supplement: Supplementary file 1 — Additional file 1. Medical School Curriculum Benchmark Survey; Description of data: Survey conducted among medical school directors. [file 12909_2020_1925_MOESM1_ESM.docx]

**Additional File 1.** **Medical School Curriculum Benchmark Survey**

**SECTION S: sample PRELOAD AND SCREENING QUESTIONS**

**ALL RESPONDENTS**

**P1 Institution from target list**

[PROGRAMMER NOTE]

IF A VALIDATED RESPONDENT FROM THE SAME INSTITUTION HAS ALREADY COMPLETED THE SURVEY, DISPLAY “Thank you for your interest in this survey. Unfortunately, someone from your institution has already participated in this survey and we are currently limiting responses to one per institution. You may receive survey invitations from us for future studies on relevant topics.”

**ALL RESPONDENTS**

**S1** Thank you for your interest in this survey. We appreciate your willingness to participate in this important research on obesity medicine education.

Before participating, KJT Group requires you to review the following information:

- Dr. Scott Butsch of the Bariatric and Metabolic Institute at the Cleveland Clinic, in collaboration

with Novo Nordisk Inc., is conducting this survey **to better understand the state of obesity**

**education in undergraduate medical education in the United States**.

- KJT Group is a **global market research company**.
- You have been invited to participate as you may be familiar with the medical student curriculum, including where and how obesity is taught.
- Your participation involves completing this survey.
- We expect, on average, it will take respondents like yourself **10 minutes** to complete this survey.
- Findings of this research project may be published in scientific journals or presented at medical

meetings. All the results will be presented in aggregate form; data will never be presented in a way

that identifies individual institutions.

- Your responses will be kept **strictly confidential** and will never be associated with your name.
- Your **participation is voluntary** and you may choose to stop participating at any time (withdraw consent).

Do you consent to these terms and wish to continue with the survey?

1. Yes [CONTINUE]
2. No [TERMINATE]

[IF AGREES TO DISCLOSURE STATEMENT (S1/1), ASK S1A. ALL OTHERS TERMINATE.]1A

**ALL RESPONDENTS**

**S1A**

In the event that we may need to contact you related to this survey, please provide your email address. If you prefer not to provide this information, please check the box below. This information will only be used by KJT Group for the sole purpose of this survey and will not be used for any marketing activities or for any other purpose.

Email address [MANDATORY TEXT BOX]

99. I prefer not to provide my email address

**ALL US RESPONDENTS**

**S3** What is your role in undergraduate medical education?

1. Course/Curriculum coordinator
2. Dean of medical education/curriculum (including assistant, associate, senior, and vice deans)
3. Curriculum director
4. Administrator
5. Other; please specify: [MANDATORY TEXT BOX]

**ADMINISTRATOR (S3/4)**

**S3A** Please describe your role as an administrator:

[MANDATORY TEXT BOX]

**ALL RESPONDENTS IN US**

**S4** How knowledgeable are you with the four-year curriculum at your medical school?

1. Very
2. Somewhat
3. A little
4. Not at all

[IF AT LEAST A LITTLE KNOWLEDGABLE (S4/1-3), ASK S5. ALL OTHERS TERMINATE.]

**AT LEAST A LITTLE KNOWLEGABLE (S4/1-3)**

**S5** What best describes the design of curriculum at your medical school? Select one of the options below.

1. Integrated organ-system-based curriculum
2. Traditional curriculum (basic science x2 years + clinical science x2 years)
3. Other; please describe: [MANDATORY TEXT BOX]

[IF TRADITIONAL CURRICULUM (S5/2), ASK S6. ALL OTHERS CONTINUE.]

**TRADITIONAL CURRICULUM (S5/2)**

**S6** How knowledgeable are you with each of the following at your medical school?

[DISPLAY AS GRID]

[COLUMNS]

1. Very
2. Somewhat
3. A little
4. Not at all

[ROWS]

1. Basic science curriculum
2. Clinical science curriculum

[IF NOT AT ALL KNOWLEDGABLE FOR BOTH BASIC AND CLINICAL (S6_r1/4 AND S6_r2/4), TERMINATE. ALL OTHERS CONTINUE]

**ALL RESPONDENTS**

**S100 FINAL QUOTA QUESTION** [n=100]

- Reside in US
- At least a little knowledgeable about 4-year medical school curriculum
- At least a little knowledgeable about basic science or clinical science curriculum if have traditional curriculum

1. Not qualified [n=9999]

**SECTION 200: Medical school curriculum assessment**

**ALL QUALIFIED RESPONDENTS**

**Q202** How long have you been at your current institution?

[RANGE 0-50]

[_|_] years

**ALL QUALIFIED RESPONDENTS**

**Q203** How long have you been in your current role as it is related to undergraduate medical education?

[RANGE 0-50]

[_|_] years

**ALL QUALIFIED RESPONDENTS**

**Q204** Do you teach medical students (at any level)?

1. Yes
2. No

**ALL QUALIFIED RESPONDENTS**

**Q205** Please indicate which of the following describes your role in undergraduate medical education:

*Please select all that apply.*

[RANDOMIZE]

1. I teach a course to medical students
2. I give lectures to medical students
3. I participate as a tutor to medical students
4. I participate as a preceptor in a clinic to medical students
5. Other; please specify: [ANCHOR] [MANDATORY TEXT BOX]

**ALL QUALIFIED RESPONDENTS**

**Q206** How long have you been involved in undergraduate medical education (total time during career)?

[RANGE 0-50] [CANNOT BE LESS THAN Q203]

[_|_] years

**ALL QUALIFIED RESPONDENTS**

**Q207** How prepared do you think graduating students at your medical school are to manage patients with obesity?

1. Very prepared
2. Fairly prepared
3. Somewhat prepared
4. Not at all prepared

**ALL QUALIFIED RESPONDENTS**

**Q208** Is obesity education taught as a standalone course at your institution (i.e., separate from general courses in clinical nutrition or lifestyle medicine)?

1. Yes
2. No
3. I don’t know

**ALL QUALIFIED RESPONDENTS**

**Q209** Is obesity education integrated into a broader clinical nutrition course or preventative medicine course?

1. Yes
2. No
3. I don’t know

**ALL QUALIFIED RESPONDENTS**

**Q210** Is obesity education taught as a case in other clinical courses?

1. Yes
2. No
3. I don’t know

**ALL QUALIFIED RESPONDENTS**

**Q211** Is obesity education taught as a case in other non-clinical courses?

1. Yes
2. No
3. I don’t know

**ALL QUALIFIED RESPONDENTS**

**Q212** Is obesity education taught as a clinical rotation in an outpatient medical clinic?

1. Yes
2. No
3. I don't know

**ALL QUALIFIED RESPONDENTS**

**Q213** Are you, **or someone else**, currently developing obesity education into your curriculum?

1. No, we already have an obesity education program in place
2. Yes, we are having active discussions on how to incorporate this topic and/or currently developing an obesity education program into our curriculum
3. No, there is currently no program offered and no plans to develop one

**Q214** To what extent is expanding obesity education a priority for your department/program?

1. High priority
2. Medium priority
3. Low priority
4. Not a priority

**ACTIVELY DEVELOPING OBESITY CURRICULUM (Q213r2)**

**Q214A** Which of the following best describes when you expect to implement or expand your obesity education curriculum?

1. Within the next year
2. 1-2 years from now
3. More than 2 years from now

**ALL QUALIFIED RESPONDENTS**

**Q215** For each of the following factors, please indicate to what degree it acts as a barrier to integrating obesity

education into your curriculum.

[COLUMNS]

1. Large barrier
2. Moderate barrier
3. Small barrier
4. Not a barrier

[RANDOMIZE] [ROWS]

1. Lack of student interest
2. Lack of faculty interest
3. Lack of room in the curriculum
4. Lack of faculty expertise
5. Financial

**ALL QUALIFIED RESPONDENTS**

**Q216** To the best of your knowledge, how many courses in the four-year curriculum have a lecture

specifically on obesity?

[RANGE 0-20]

[_|_] courses

99. I don’t know

**ALL QUALIFIED RESPONDENTS**

**Q217** To the best of your knowledge, how many courses in the four-year curriculum have a clinical case

specifically on obesity?

[RANGE 0-20]

[_|_] courses

99. I don’t know

**ALL QUALIFIED RESPONDENTS**

**Q218** To the best of your knowledge, how many TOTAL hours of the four-year curriculum are dedicated to

obesity education?

[RANGE 0-50]

[_|_] hours

99. I don’t know

**ALL QUALIFIED RESPONDENTS**

**Q219** The following are considered core obesity competencies. For each competency, please indicate the extent

each is included in the curriculum at your medical school.

[DISPLAY AS CAROUSEL; ONE ROW PER SCREEN.]

[COLUMNS]

1. Great extent
2. Some extent
3. Very little
4. Not at all

99. I don’t know

[PROGRAMMER NOTE] PLEASE DISPLAY c99 AS A SEPARATE RESPONSE THAT CAN BE SELECTED FOR EACH ROW]

[RANDOMIZE] [ROWS]

1. Knowledge of obesity epidemiology (i.e., common environmental, social, and behavioral contributors to the obesity epidemic; incidence and prevalence of obesity, etc.)
2. Knowledge of energy homeostasis and weight regulation (i.e., physiology of obesity and related hormones, e.g., ghrelin, leptin, POMC, NPY, etc.)
3. Knowledge of body composition measurements (e.g., waist circumference, bioimpedance analysis, waist-to-hip ratio) and clinical assessment tools of energy expenditure (e.g., indirect calorimetry)
4. Knowledge of the etiologies, mechanisms, and biology of obesity (including genetic forms of obesity)
5. Knowledge of obesity-related comorbidities and corresponding benefits of weight loss
6. Knowledge of nutrition interventions (e.g., calorie balance, macronutrients, energy density) to develop a comprehensive obesity management care plan
7. Knowledge of physical activity (e.g., aerobic, resistance training, exercise prescriptions) to develop a comprehensive obesity management care plan
8. Knowledge of behavioral interventions (e.g., cognitive behavioral therapy) to develop a comprehensive obesity management care plan
9. Knowledge of evidence-based models of behavior change (e.g., motivational interviewing) to effectively assess patients’ readiness for weight management counseling
10. Knowledge of pharmacological treatments (i.e., recognize the six FDA-approved anti-obesity medications as an appropriate form of therapy) as part of a comprehensive obesity management care plan
11. Knowledge of appropriate language usage in verbal, non-verbal, and written communication that is non-biased, non-judgmental, respectful, and empathetic when communicating with patients with obesity
12. Knowledge of a comprehensive obesity-focused medical history (including questions on exercise, diet, sleep, stress) from a patient with obesity
13. Knowledge of a comprehensive physical exam (including use of size-appropriate gowns, BP cuffs, etc.) in patients with obesity
14. Knowledge of policies and public health initiatives pertaining to obesity

**ALL QUALIFIED RESPONDENTS**

**Q220** Does your institution use virtual learning systems (e.g., web-based programs such as Nutrition in

Medicine™ modules, etc.) to provide obesity education?

1. Yes
2. No
3. I don’t know

**ALL QUALIFIED RESPONDENTS**

**Q221** Does your institution offer elective shadowing opportunities with non-medical providers (e.g., dietitians,

psychologists, etc.) who specifically treat patients with obesity?

1. Yes
2. No
3. I don’t know

**ALL QUALIFIED RESPONDENTS**

**Q222** Are there formal surgical clinical rotation opportunities in obesity (e.g., bariatric surgery clinic) for

students?

1. Yes
2. No
3. I don’t know

**ALL QUALIFIED RESPONDENTS**

**Q223** Are there bariatric surgeons at your institution?

1. Yes
2. No
3. I don’t know

**ALL QUALIFIED RESPONDENTS**

**Q224** Is there a board-certified obesity medicine specialist (a physician who has a certification in obesity

medicine from the American Board of Obesity Medicine) on your faculty?

1. Yes
2. No
3. I don’t know

| **Section 100: Demographics** |
| --- |

**ALL QUALIFIED RESPONDENTS**

**Q100** How many undergraduate medical students are currently enrolled at your institution?

*Your best estimate will do.*

[RANGE 1-3000]

[_|_] undergraduate medical students

99. I don’t know

**ALL QUALIFIED RESPONDENTS**

**Q101** How many faculty teach undergraduate medical education at your institution? Please consider faculty with any title and those who teach part time or full time.

*Your best estimate will do.*

[RANGE 1-700]

[_|_] faculty

99. I don’t know

**ALL QUALIFIED RESPONDENTS**

**Q102** Is your institution public or private?

1. Public
2. Private
3. I don’t know

**ALL QUALIFIED RESPONDENTS**

**Q103** Is your institution considered a Bariatric Center of Excellence?

1. Yes
2. No
3. I don’t know
